# Three-dimensional characteristics of the alveolar capillary network in infant and adult human lungs

**DOI:** 10.1038/s41390-024-03572-y

**Published:** 2024-09-23

**Authors:** Giacomo Rößler, Jonas Labode, Julia Schipke, Stefan A. Tschanz, Christian Mühlfeld

**Affiliations:** 1 Institute of Functional and Applied Anatomy, Hannover Medical School, Hannover, Germany; 2 Biomedical Research in Endstage and Obstructive Lung Disease Hannover (BREATH), Member of the German Center for Lung Research (DZL), Hannover, Germany; 3 Institute of Anatomy, University of Bern, Bern, Switzerland; 4 Research Core Unit Electron Microscopy, Hannover Medical School, Hannover, Germany

## Abstract

**Background:**

A comprehensive understanding of vascular development in the human lung is still missing.

**Methods:**

Therefore, samples of infant (*n* = 5, 26 days to 18 months postnatally) and adult (*n* = 5, 20 to 40 years) human lungs were subjected to unbiased stereological estimation of the total number of capillary loops. Serial sections were segmented to visualize the alveolar capillary network (ACN) in 3D.

**Results:**

The number of capillary loops increased in parallel to lung volume from 26 days to 18 months, while in adults, it was not correlated to lung volume. In infant lungs, two capillary layers were separated by a connective tissue sheet with a growing number of interconnections. In adults, the mature ACN was almost, but not completely, single-layered. Here, the connective tissue was thinner but still centrally positioned, suggesting the persistence of interconnected parts of both layers of the previously double-layered ACN.

**Conclusions:**

Small parts of the capillaries remain double-layered and seem to be grouped around the thin connective tissue sheet, suggesting a different mechanism of microvascular maturation than simple fusion of the two layers. These spots are a potential basis for further alveolarization after completion of bulk formation.

**Impact:**

- The 3D data offer a new conceptual approach to microvascular maturation of the lung.
- Microvascular maturation rather results from reduction than simple fusion of capillary fragments.
- Adult lungs maintain small double-layered capillary spots.
- These could offer a potential source of regeneration.
- The data are important to better understand normal and pathological lung development.

## Introduction

When mammals are born, their lungs are designed to function as a gas exchanger, i.e., the alveolar and capillary surface area need to be large and the air-blood barrier thin enough to allow sufficient gas exchange to meet the increased physiological demands of extrauterine life.^1^ Although, in principle, mammalian lung development follows a similar pattern of four subsequent, but overlapping postembryonic periods, the developmental stage an animal is born in depends on the species.^2^ The different stages are termed according to the histological occurrence of characteristic morphological features: pseudoglandular, canalicular, saccular and alveolar.^3^ Rodents, for example, are born relatively immature before the onset of the alveolar stage. In humans, alveolarization starts already before birth at 36 weeks after conception and continues through the first years of postnatal life with most alveoli being formed until the age of two years, so-called bulk alveolarization.^4–6^ After that, new alveoli are formed to a much smaller extent (continued alveolarization) and the lung mainly grows by enlargement of the existing airspaces. Thus, postnatal lung development can be regarded as a biphasic process in mammals.^7^

The formation of new alveoli is a well-coordinated process that involves not only the alveolar epithelium but also the capillary network within alveolar septa. In the immature lung, the septa contain a central connective tissue sheet with capillaries on both sides, the double-layered capillary network. During alveolarization the two layers are transformed to a single layered capillary network, a process called microvascular maturation. The double layered capillary network is considered to be important for the formation of new alveolar septa during bulk alveolarization and the transition to a single layer marks the end of this phase. Recent evidence suggests that during the age of 1 to 3 years in humans most of the microvascular maturation has taken place, thus leading into the phase of continued alveolarization.^8^

So far, little information on the 3D characteristics of the microvascular maturation process is available. In the last years, however, new imaging and analytical tools have been developed that allow both three-dimensional visualization and quantitative assessment of lung development and the capillary network.^9,10^ These methods include radiological^11^ and (electron) microscopic techniques^12–14^ as well as stereological estimation of the number of capillary loops.^15^ Three-dimensional reconstructions of postnatal mouse lungs after exposure to hyperoxia have demonstrated a disruption of the double-layered capillary network in combination with reduced alveolarization.^12^ Furthermore, in the neonatal mouse, it was recently shown using serial block-face scanning electron microscopy that the two sheets of the double-layered capillary network are not strictly separate sheets but rather form a three-dimensional network with multiple connections between both layers already on the first day of postnatal life.^16^ However, in human lungs interconnections between the two networks were described only to be rarely present at one month after birth, but were more frequently observed at 6 months.^5^ Whether the two sheets are interconnected already early after birth or nearly completely separated requires a 3D analysis. In addition, Zeltner and Burri^5^ stated that even in adult human lungs sometimes a double-layered capillary network appeared to be present, but that this might also be due to the cutting angle. Thus, a 3D reconstruction is needed to clarify whether parts of the alveolar capillaries are still organized in two layers in adult human lungs. Therefore, the present study made use of serial sections of human lung samples to answer these questions. Furthermore, stereology was used to estimate the number of capillary loops in infant and adult human lungs, and to correlate these data with the increases in lung volume as well as capillary surface area and septal blood volume. The results of the quantitative and 3D analyses were intended to shed light on the growth characteristics of the postnatal human lung during alveolarization and microvascular maturation.

## Methods

### Origin of human lung samples

The current study was performed on archived lung tissue originating from the studies performed by Zeltner et al.^17^ on infant and Gehr et al.^18^ on adult lungs, respectively. The fixation and use of the lungs was according to the bioethical regulations of the University of Bern at that time. The use of the archived material was approved by the ethics committee of Hannover Medical School (Permission No. 2263-2014). The adults from which the lungs were obtained died from serious cerebral injury or from cardiac arrest, the infants died from isolated head injury, intracerebral haemorrhage, sudden infant death syndrome and acute viral encephalitis (for details see Zeltner et al.^17^ and Gehr et al.^18^). In compliance with current standards for human subjects, the lungs were deidentified by assigned numbers. Five adult and five infant lungs of the originally reported ones were arbitrarily picked for this study.

After confirmed death, both lungs were fixed by airway instillation of a 2.5% glutaraldehyde and phosphate buffer solution. Through stratified random sampling schemes, several regions were selected for following analyses. The samples used in this study were subsequently incubated with osmium tetroxide and uranyl acetate before dehydration and embedding in epoxy resin. For further details see Zeltner & Burri (1987)^5^, Zeltner et al. (1987)^17^ and Gehr et al. (1978)^18^.

### Design-based stereology

For stereological analyses, histological samples were stained with toluidine blue and digitalized using an Axio Scan.Z1 (Zeiss, Göttingen, Germany) slide scanner at an objective lens magnification of 40x. All stereological methods were applied in accordance with the recommendations for quantitative assessment of lung structure by the American Thoracic Society/European Respiratory Society^19^ and based on standards of design-based stereology.^15,20,21^ Stereological analyses were executed with the newCAST™ software (Visiopharm®, Hørsholm, Denmark). Automated systematic uniform random sampling (SURS)^22^ was used as a main principle to ensure nonoverlapping, random fields of view for each parameter. To ensure registration of at least 200–300 counting events for each parameter per lung, sampling fractions were set between 20–40%.

Estimation of surface density and total surface area of the endothelium as well as estimation of numerical density and total number of septal capillary loops (SCL) was performed at a 40x objective lens magnification as described in detail by Rößler et al.^23^. In brief, for estimating the endothelial surface area, we used a 52.9 μm long line probe and counted the number of intersections with the endothelium. The total surface area was calculated by multiplying the surface density with the volume of the reference space.

To quantitatively characterise the complexity of the ACN, we utilised the Euler–Poincaré characteristic (EPC) as previously introduced for this context by Willführ et al.^15^ to estimate the total number of SCL. A SCL consists of a cycle of capillary segments that surrounds one tissue pillar. Using a physical disector, the connectivity of the septal capillaries can be evaluated by counting of three topological events: a “bridge” describes a new connection between two capillary profiles, i.e. two separate capillary profiles in one section are connected in the other section; an “island” is the occurrence of an isolated capillary profile that is not present in the other section; the term “hole” describes that an enclosed cavity within a capillary profile is present in one section but not in the other.^15,23,24^

Using the newCAST™ software, two consecutive tissue sections were aligned and a physical disector of 1 μm disector height was employed. An unbiased counting frame with an area of 10,000 μm^2^ was projected on the randomly sampled test fields. The numerical densities of SCL were calculated by dividing the Euler number χ by the total disector volume multiplied by two for counting events on both sides of the image pairs. Multiplying numerical densities by their corresponding reference space volume produced the total numbers of SCL.

Lung volume data, assessed by water displacement method, and volume of parenchyma were adopted from the original investigations.^17,18^

### 3D analysis

Serial sections of two young infant (I1 and I2), three older infant (I3-I5) and three adult (A1, A3, A4) lungs were analysed.

From three different lungs (I2: 30 days, I4: 17 months, A1: 20 years), 3D reconstructions of a portion of the ACN in relation to tissue and airspace were created to visualize the major findings.

**Fig. 1:**
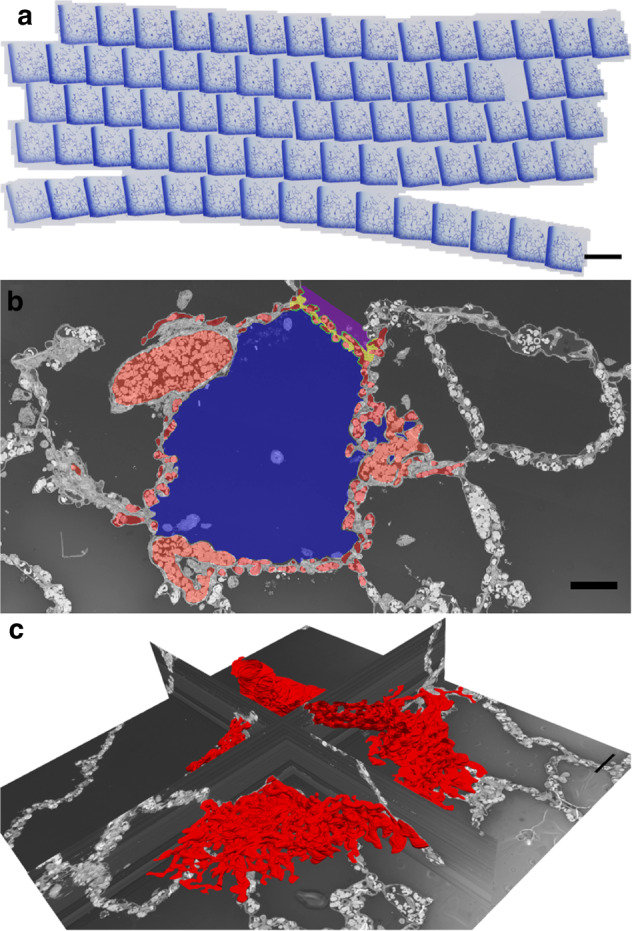
From serial sections to the 3D model. **a** Serial sections of the parenchymal region were obtained from a 20-year-old lung and stained with toluidine blue (also done for a 30-day- and a 17-month-old lung). Scale bar = 2 mm. **b** The digitalized sections were segmented manually and colour-labelled. Red = vasculature; yellow/green = septal interstitium; blue/purple = airspace lumen; scale bar = 50 µm. **c** The 3D model of the vasculature (red) can be viewed in relation to the 2D representation. As such, it is possible to analyse the model by referring to the 2D sections. Scale bar = 50 µm.

For each lung, a consecutive row of 175 semi-thin sections with 0.5 μm thickness was created. The section thickness was chosen to allow a similar resolution in x, y and z direction and to avoid information being lost between two sections, for example, due to oblique cutting angles of capillary segments. The series of sections were placed on glass slides, stained with toluidine blue and digitalized using an Axio Scan.Z1 (Zeiss, Göttingen, Germany) slide scanner at a primary magnification of 40x (Fig. 1a). An adapted version of procedures established by Grothausmann et al.^13^ and Grothausmann et al.^25^ was used to create a 3D dataset from each series of histological sections. In short, the centre of every section was manually assigned using Fiji’s (v1.52p)^26^ multi-selection tool, then individual sections were automatically cropped, extracted and converted to 8-bit grey images. Consecutive images were stacked and aligned to their neighbours, restoring the third (z-) dimension and thus recreating the physiological spatial arrangement. This virtual image registration was achieved by the Python script recRegStack.py (http://github.com/romangrothausmann/elastix_scripts/) based on SimpleElastix^27^. The result was a 3D image stack with an isometric voxel size of 0.5 μm, equivalent to the thickness of the original sections.

The tissue was unsuitable for automated segmentation procedures, because the performed instillation method resulted in residing erythrocytes in capillaries and non-capillary vessels. Therefore, the time-consuming but more precise method of manual segmentation was chosen, i.e. ITK-SNAP’s (v3.4.0)^28^ Paintbrush mode to label the endothelial lumina, airspace and interstitium manually (Fig. 1b). This resulted in a 3D reconstruction of the ACN and its neighbouring interstitial structures in relation to the airspace (Fig. 1c).

### Axial skeleton of the ACN

Additionally, the 3D dataset of the microvasculature was converted to a dot graph using SGEXT^29^, i.e. reducing the volume rendering of capillaries (voxels) to an axial skeleton of the ACN (vectors), consisting only of branches (edges) and branching points (nodes). This simplified model of the ACN retains the topology of the original segmentation and does not change the Euler number χ. The dot graph was manually cleaned from erroneous or isolated edges and nodes.

For visualization, the dot graph was converted to the 3D data file format VTU using a Python script (https://gitlab.gwdg.de/jonas.labode/dot2vtu) and then merged with the 3D model using Paraview (v5.8.0)^30^. The open-source programs Paraview and Fiji were further used to render, arrange and annotate the 3D datasets to produce the presented figures. (Fig. 1).

### Digital assessment of capillary central connective tissue thickness

To assess the dimensions of segmentation (i.e. thickness of capillaries and thickness of the central connective tissue sheet), Fiji’s Local thickness plugin^26^ was used. This tool measures the local thickness of the examined structure in every point and computes this data into a visual Distance map, depicting the distance to the boundaries of the structure. The Python script Mean Image Value (https://github.com/labode/mean_image_value) was applied to extract the mean pixel value of the Distance map, resulting in the mean capillary thickness of the ACN and the mean thickness of the central connective tissue sheet, respectively. The results, scaled in voxels, were multiplied by 0.5 (voxel size) to obtain the biological scale in µm.

### Statistics

Correlation analyses were performed by one-tailed Spearman’s rank correlation. The correlation between two variables was considered significant when *p* < 0.05. The relationship between the variables was described by the power law equation y = ax^b^.

## Results

### Stereological data

Table 1 summarizes the stereological data obtained from the infant and adult human lungs.

**Table 1:** Basic parameters and stereologically acquired data on septal capillaries of the lung.

	I1	I2	I3	I4	I5	A1	A2	A3	A4	A5
Age	26 d pp	30 d pp	16 mt pp	17 mt pp	18 mt pp	20 y	22 y	35 y	39 y	40 y
Sex	m	m	f	f	m	m	m	f	m	m
W, kg	3.04	4.1	8.8	10	12.5	72	96	76	85	70
V (lung), ml	142	192	480	504	714	3900	3800	4600	5950	3650
V (par,lung), ml	115	157	400	406	627	3510	3420	4140	5355	3285
N_V_ (cap/par), x10^−5^ μm^−3^	3.112	4.552	2.825	6.2	3.282	5.354	3.779	3.755	2.248	2.528
N (cap,lung), x10^9^	3.57	7.167	11.307	25.175	20.572	187.912	129.248	155.456	120.375	83.039
N (cap,lung) / W, x10^9^/kg	1.174	1.748	1.285	2.517	1.646	2.610	1.346	2.045	1.416	1.186
N (cap,lung) / V (lung), x10^9^/ml	0.025	0.037	0.024	0.05	0.029	0.048	0.034	0.034	0.020	0.023
S_V_ (endo/par), μm^−1^	0.022	0.034	0.02	0.042	0.02	0.034	0.025	0.021	0.017	0.020
S (endo,lung), m^2^	2.497	5.375	8.136	17.099	12.494	119.752	87.024	88.550	88.469	64.566
S (endo,lung) / W, m^2^/kg	0.821	1.311	0.925	1.71	0.999	1.663	0.907	1.165	1.041	0.922
S (endo,lung) / V (lung), m^2^/ml	0.018	0.028	0.017	0.034	0.017	0.031	0.023	0.019	0.015	0.018

*W* body mass, *V (lung)* lung volume, *V (par,lung)* parenchymal volume, *N*_*V*_ *(cap/lung)* numerical density of septal capillary loops, *N (cap,lung)* number of septal capillary loops, *SV (endo/lung)* surface density of capillaries, *S (endo,lung)* capillary surface area, *d* days, *mt* months, *y* years, *pp* postpartum, *m* male, *f* female. Body mass, lung volume and parenchymal volume were taken from Zeltner et al.^17^ for the infant and from Gehr et al.^18^ for the adult lungs.

The relative amount of capillaries per unit of parenchymal volume was in a relatively narrow range and did not show any systematic developmental pattern over time. The number of capillary loops per unit volume of parenchyma (N_V_ (cap/par)) ranged between 2.2 × 10^−5^/µm³ in the 39 y old lung and 6.2 × 10^−5^/µm³ in the 17 mt old lung. Similarly, the surface density of capillary endothelium per unit parenchyma volume (S_V_ (endo/par)) ranged from 0.017 µm^−1^ to 0.042 µm^−1^ with the 39 y old and the 17 mt again being the minimum and maximum, respectively. When the number of capillary loops and the surface area of the endothelium were related to body weight (N (cap,lung)/W and S (endo,lung)/W, respectively) or to total lung volume (N (cap,lung)/V (lung) and S (endo,lung)/V (lung), respectively) the range between the infant and adult lungs was similarly small with the minima and maxima being differently distributed among the individuals. This is not surprising because both lung volume and body weight are less stable reference volumes than parenchyma due to other influences such as inflation or alimentary status. The total number of capillary loops (N (cap,lung)) increased from 3.57 × 10^9^ in the 26 d old infant to 187.9 × 10^9^ in the 20 y old adult. The infants between 16 and 18 months old had a total number of capillary loops between 11 and 25 × 10^9^. Remarkably, there seemed to be a decline of the total number of capillary loops over time in the adult lungs. The total surface area of the endothelium (S (endo,lung)) showed a similar pattern with 2.5 m² in the 26 day old and 120 m² in the 20 year old individual.

**Fig. 2:**
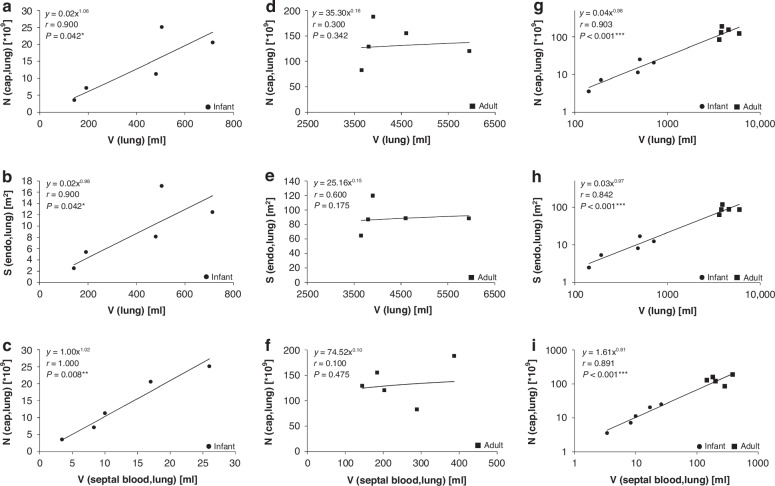
Correlation analysis of stereological data. Correlation analyses were performed by one-tailed Spearman’s rank correlation. The correlation between two variables was considered significant when *p* < 0.05. The relationship between the variables was described by the power law equation y = ax^b^. Note the double logarithmic scale when infant and adult data were plotted together. *N*(Cap) = Number of capillary loops; S(Endo) = Surface area of capillary endothelium; V(lung) = Volume of lung. The data of septal blood volume were taken from Zeltner et al.^17^. Correlation of number of capillaries with lung volume in infants (**a**), in adults (**d**) and in infants and adults (**g**). Correlation of endothelial surface area with lung volume in infants (**b**), in adults (**e**) and in infants and adults (**h**). Correlation of number of capillaries with septal blood volume in infants (**c**), in adults (**f**) and in infants and adults (**i**).

Plotting of number and surface area versus lung volume and septal blood volume shows remarkable differences when infants and adults are plotted separately or together. In Fig. 2 the left (a–c) and middle (d–f) column show the separate plotting of infant and adult individuals, respectively, whereas the right (g–i) column displays the scenario of infants and adults together. When plotting the number and surface area of the capillary loops in relation to lung volume separately for infants and adults, there is a clear dependence of these parameters on lung volume during postnatal development, but not in the adult lung. Both parameters scale with lung volume during infancy significantly with an exponent close to 1 (Fig. 2a, b) whereas there is no such correlation in the adult lungs (Fig. 2d, e). This suggests that the lung volume increase in early postnatal life is mainly due to the addition of new structural components, whereas the differences in adult lung volume are not proportional to the amount of structural components. Nevertheless, both number and surface area of the capillaries increased between the 16- to 18-month-old infant lungs and the adult lungs by a mean factor of approximately 7 (Table 1). This means that although microvascular maturation, the transition from a double-layered to a single-layered capillary network, takes place, further addition of new microvascular elements is required during development from infancy to young adulthood. Both number of capillary loops and endothelial surface area scaled with an exponent of approximately 1 with lung volume when infants and adults were plotted together (Fig. 2g, h) which emphasizes a relatively fixed amount of microvasculature per unit of lung volume irrespective of the underlying developmental processes. Interestingly, a correlation between the septal blood volume (data taken from Zeltner et al.^17^) and the number of capillary loops shows a remarkable difference when infants and adults are plotted together or separately. When using the power law equation y = ax^b^ for infants and adults separately one finds that b is close to 1 for infants (Fig. 2c) and only 0.1 for adults (Fig. 2f). However, when the equation is calculated for infants and adults together *b* = 0.81 (Fig. 2i). This means that in infant lungs the number of capillary loops per unit of the septal blood volume is higher than in adults which fits nicely with the concept of the double-layered capillary bed and the microvascular maturation.

### 3D analysis

**Fig. 3:**
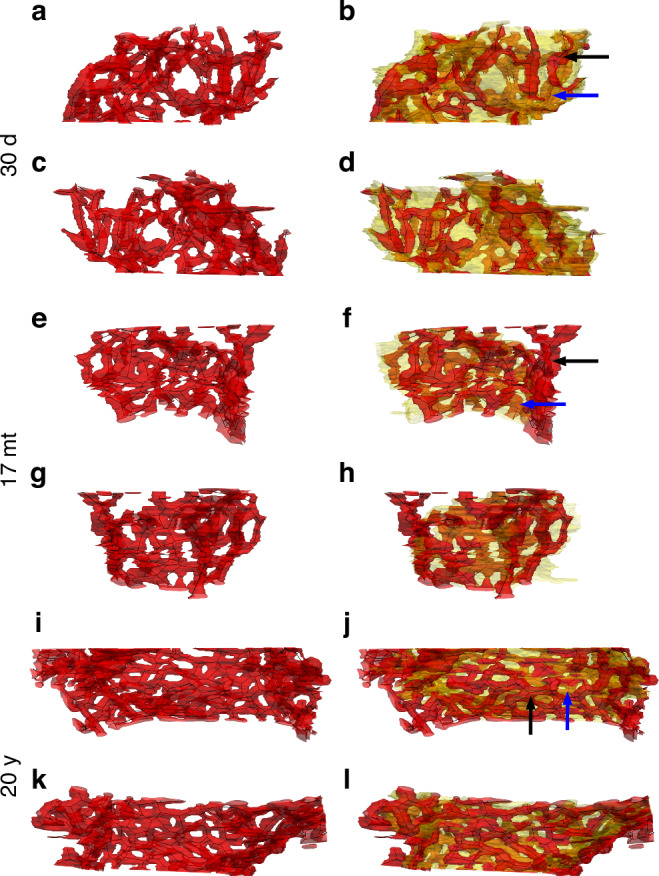
Overview of the ACN models shown in detail in Figs. 4 to 6. **a**–**d**: 30-day-old infant. **a**, **c** show the same model of the ACN (red) from the front and back, respectively. In **b**, **d** the interstitial layer (transparent yellow) is added. **e**–**h** 17-month-old infant. **e**, **g** show the same model of the ACN (red) from the front and back, respectively. In **f**, **h** the interstitial layer (transparent yellow) is added. **i**–**l**: 20-year-old adult. **I**, **k** show the same model of the ACN (red) from the front and back, respectively. In **j**, **l** the interstitial layer (transparent yellow) is added. Dimmed red areas show capillaries that lie within or behind the connective tissue sheet (examples labelled by blue arrows). Bright red areas indicate capillaries in front of the connective tissue sheet (examples labelled by black arrows). A system of black dots and connecting lines illustrates the axial skeleton of the ACN. Similar perspectives were chosen for the three cases.

The 3D analysis was performed on serial sections from young infant (*n* = 2), appr. 17 months old infant (*n* = 3) and adult (*n* = 3) lungs. Segmentations were performed on small areas from one lung per age group to highlight the 3D observations. Figure 3 provides an overview of the 3D characteristics of the capillary network in a 30-day-old and a 17-month-old infant as well as a 20-year-old adult. For each individual, the obtained model of the ACN (red) is shown from two sides without (left column) and with (right column) the connective tissue sheet (transparent yellow) of the septum. In addition, the axial skeleton of the capillaries is shown as black lines. Dimmed red areas show capillaries that lie within the septum or on the opposite side. Bright red areas indicate the air-blood-barrier (corresponding to thin regions of the septum). The overview demonstrates that already 30 days after birth the layers of the ACN are not strictly separated, but that there are interconnections between the two layers, even if they are not yet frequent. The number of interconnections becomes more pronounced at 17 months, and the ACN looks denser. At both 30 days and 17 months of age, the capillary loops have not yet reached the hexagonal appearance with 6 branching points, capillary loops with 4 or 5 branching points can often be seen. In the adult lung, the ACN is denser and has a much higher degree of regularity, and most capillary loops are hexagonal in shape.

**Fig. 4:**
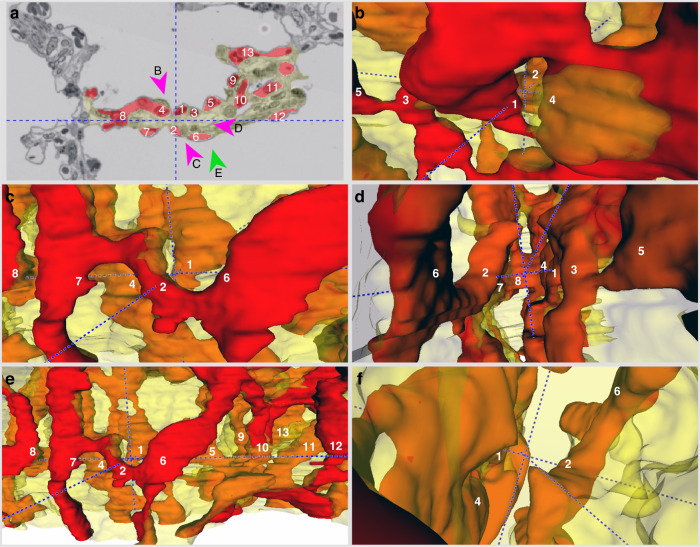
3D visualization of the ACN in a 30-day-old infant. **a** 2D section from the serial sections used for segmentation. The red labelling depicts capillaries, the yellow labelling the septal interstitium. Individual capillary profiles are marked by numbers for identification and orientation in the 3D model. The dashed lines mark the orthogonal crosshairs of the model (same location in all images (**a**–**f**)). Arrowheads with the letters **b**–**e** mark the angle from which the 3D visualization is shown in the respective images. On average, capillaries in this segmentation had a diameter of 4.47 µm and the connective tissue sheet a thickness of 7.88 µm. Scale bar = 15 µm. **f** Top view into the 3D model.

**Fig. 5:**
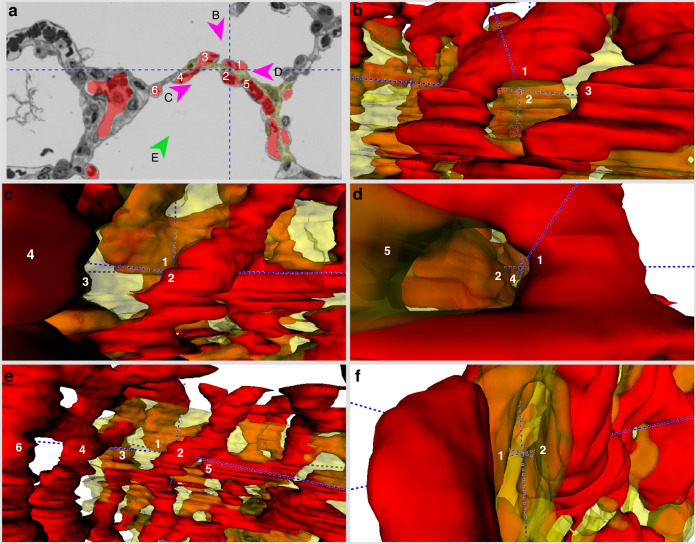
3D visualization of the ACN in a 17-month-old infant. **a** 2D section from the serial sections used for segmentation. The red labelling depicts capillaries, the yellow labelling the septal interstitium. Individual capillary profiles are marked by numbers for identification and orientation in the 3D model. The dashed lines mark the orthogonal crosshairs of the model (same location in all images (**a**–**f**)). Arrowheads with the letters **b**–**e** mark the angle from which the 3D visualization is shown in the respective images. On average, capillaries in this segmentation had a diameter of 3.73 µm and the connective tissue sheet a thickness of 3.34 µm. Scale bar = 15 µm. **f** Top view into the 3D model.

**Fig. 6:**
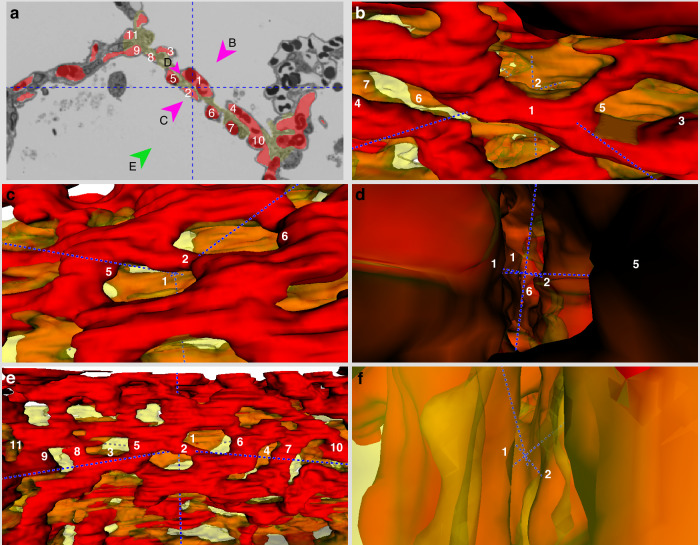
3D visualization of the ACN in a 20-year-old adult. **a** 2D section from the serial sections used for segmentation. The red labelling depicts capillaries, the yellow labelling the septal interstitium. Individual capillary profiles are marked by numbers for identification and orientation in the 3D model. The dashed lines mark the orthogonal crosshairs of the model (same location in all images (**a**–**f**)). Arrowheads with the letters **b**–**e** mark the angle from which the 3D visualization is shown in the respective images. On average, capillaries in this segmentation had a diameter of 4.5 µm and the connective tissue sheet a thickness of 3.65 µm. Scale bar = 15 µm. **f** Top view into the 3D model.

Figures 4–6 provide more detailed views of the ACN models with one two-dimensional (2D) image taken from the z stack and the 3D visualization of the model from various angles. The 2D image contains the segmentation for capillaries (red) and for connective tissue (transparent yellow) as well as numerical identifiers of the capillaries to identify them in the 3D model. To enhance the orientation, the blue dashed line shows the orthogonal xyz axes of the models. In addition, the different viewpoints from which the 3D models are visualized are indicated in the 2D sections.

In the 30-day-old lung, the central sheet separating the two layers of the ACN is relatively thick, equally thick as or even thicker than one of the capillary sheets. It separates the two sheets of the ACN nearly completely. Interconnections between the both sheets are only rarely observed, e.g. capillary no. 8 in Fig. 4. The diameter of the capillaries seems to show large variations, which is probably not a fixed morphological characteristic, but rather related to the airway and vascular pressure at the time of fixation. Also, the presence of red blood cells in the ACN influences the appearance of the ACN in the 3D visualization.

In the 17-month-old lung, the alveolar septa have become much thinner (Fig. 5). The two reasons for this are the reduction of the connective tissue sheet in the centre of the septa as well as the partial reduction of the two layers of the ACN. Connections between the two capillary layers have become more frequent, whereas parts of the septa with only single capillaries can be observed, e.g. capillaries no. 4–6 (Fig. 5). This indicates that parts of the ACN are reduced on both sides of the connective tissue.

The 20-year-old lung shows the mature ACN of the adult lung. The ACN shows a dense, regular pattern with the typical hexagonal branching of the segments (Fig. 6e). However, it can be seen that the central connective tissue sheet still divides the ACN into two layers that partially overlap and are interconnected. Where one layer is reduced, one side of the capillary is located directly under the alveolar epithelium, while the other side is adjacent to the connective tissue sheet, e.g. capillary 5 and 6 in Fig. 6. Thus, the 3D morphology suggests that during microvascular maturation, the density of the two layers of the ACN was reduced by the reduction of capillary segments, whereas the number of connections crossing the connective tissue between both capillary layers increased.

## Discussion

The present study for the first time reports unbiased quantitative data on the number of capillaries in the alveolar septa of the human infant and adult lung. These data complement the previously known functionally relevant data on volume and surface area of the ACN and allow insight into the development of the lung during alveolarization and microvascular maturation. In addition, the quantitative data are complemented with sophisticated 3D reconstructions of the ACN that help to understand the underlying processes involved in the maturation of the microvasculature of the human lung.

The first major result of the present study was the different scaling characteristics of quantitative capillary parameters in infant and adult lungs. While both number and surface area increase nearly in proportion with lung volume in infant lungs, there is no such correlation in adult human lungs. These data show that the increase in lung volume during early postnatal development depends on the generation of new vascular elements, whereas in adults, differences in lung volume are rather related to the size of the airspace. This is in accordance with other reports that considered lung development after early childhood mainly as a growth process of the existing structures.^31,32^ However, when alveoli grow, their surface area also increases which necessitates the addition of capillary segments to the ACN, which is demonstrated by the larger number of capillary loops in the adult compared with the infant lungs. Thus, despite microvascular maturation being completed to 3/4 until the age of 3 years in humans,^8^ further ACN expansion is required.

A second finding from the quantitative data is the different scaling of the number of capillaries with septal blood volume in infants alone and in infants and adults as a combined cohort. While in infants alone there is a clear proportionality between the septal blood volume and the composing elements of the ACN, the exponent in the power law equation is only 0.81 when the data of infants and adults are combined. This clearly shows that the number of capillaries per unit of blood volume they contain is higher in infants than in adults. This suggests that the size of the capillary loops is higher in adult compared to infant lungs, and that the infant lung contains an overproportionately high number of capillary loops in relation to its blood content. This underlines the function of capillaries as drivers of alveolarization during development^3,33^ beyond their role as a blood supply system.

Technical advances in imaging and segmentation for 3D visualization have led to new insights into lung development in the last 15 years.^9^ For example, the previously described septal crests could be more accurately visualized as septal ridges protruding into the airspace lumen, thus subdividing the lumen into alveolar subunits.^9,34^ It has also helped to characterize structures such as acini that cannot be delineated in 2D sections^11^ or to determine the position of pulmonary arterial branches within the vessel tree before subsequent morphometric analysis.^35–37^ Here, we used serial semithin sections of archived lung samples, which were digitized using a slide scanner and segmented by the method of manual drawing. Although the combination of serial block-face scanning electron microscopy and a semi-automated segmentation had previously facilitated the workflow,^16^ here the use of archived samples prohibited the use of serial block-face SEM, and the great number of intravascular erythrocytes made the semi-automatic approach via watershed algorithms unsuitable. This limited the tissue size that could be segmented within a reasonable time frame.

The 3D reconstruction showed that the interconnections between the two capillary layers increase during postnatal development and the existence of a central connective tissue sheet between both layers, thus confirming the findings by Zeltner and Burri^5^. In principle, the two layers of the network mainly were reduced to a single layer by the age of 20 years, a well-known process called microvascular maturation.^5,38,39^ The double-layered capillary network is considered to be essential for bulk alveolarization.^3–5^ Microvascular maturation which occurs already during the phase of bulk alveolarization marks the end of the pulmonary capacity to generate new alveoli at large scale.^40^ Interference with these processes causes disturbed vascular and alveolar development. For example, premature birth and associated conditions such as mechanical ventilation, hyperoxia or inflammation lead to a disruption of the alveolar development and a chronic lung disease named bronchopulmonary dysplasia. According to the vascular hypothesis, the injury of the ACN precedes the alteration of the alveolarization process in humans^41,42^ and experimental animals.^12,43^ In a rabbit model it was recently shown that preterm birth and hyperoxia had distinct effects on alveoli and capillaries with preterm birth having a strong effect on alveolarization and hyperoxia strongly affecting the ACN.^23^ Premature reduction of the double-layered capillary network was induced in rodents by postnatal dexamethasone treatment and was also associated with a reduced alveolarization.^39,44,45^ During microvascular maturation the two layers of the ACN are hypothesized to fuse, thus forming a single layer.^39^ The 3D morphology of the alveolar septa observed in our study indicate a different mechanism. The 3D reconstruction suggests that the central connective tissue sheet persists (while thinning) with capillary segments of both layers remaining (and vanishing) on either side of the sheet. This interpretation could also explain the importance of an increasing number of interconnections between the sheets. Studies on apoptotic cell death during postnatal development, however, rather imply that fibroblasts and alveolar epithelial cells undergo apoptosis.^46–48^

Zeltner et al.^17^ proposed that the single-layered ACN might be an idealized state that is not fully reached in the whole lung. However, this hypothesis was based on 2D sections, thus making it prone to misinterpretation of the cutting angle. Here, we provide evidence that indeed some capillary segments are still double-layered but this is restricted only to distinct areas of the ACN. Whether these parts of the ACN may act as “outgrowing regions” to form new alveolar septa in the adult lung remains doubtful. If the microvascular maturation occurred by loss of capillary segments in both layers rather than by fusion as previously suggested, this could explain why capillaries are still present on both sides of the connective tissue sheet in some parts of the septum. In summary, the present study provides unbiased reference data on the ACN of the infant and adult human lung, showing that capillary number grows with lung volume during the first two years of life, but that it is not proportional to lung volume in adulthood. The 3D reconstruction confirms the double-layered capillary network in the developing lung and the reduction to an almost single capillary network in adulthood. The mode of microvascular maturation requires further investigation as other mechanisms than fusion may act here as well, namely a loss of capillary segments in both layers.

## Data Availability

The datasets generated during and/or analysed during the current study are available from the corresponding author on reasonable request. Makefiles are available upon request.
